# 

**Published:** 1891-08

**Authors:** 


### BOOKS AND PAMPHLETS RECEIVED.

On the external characters of fœtal Reindeer and other notes, by *R. W. Shufeldt, M.D.* Reprinted from the Proceedings of the Academy of Natural Sciences. Philadelphia, 1891.

On the question of Saurognathism of the Pici and other osteological notes upon that groupe, by *R. W. Shufeldt, C.M.Z.S.* Reprinted from the Proceedings of the Zoological Society, London, February 3, 1891.

Beschreibung dreier Mikrocephalen—Gehirne nebst Vorstudien zur Anatomie der Mikrocephalie von *Dr. Felix Marchand, M.A.N.* Halle, 1890.

Journal of the Cincinnatti Society of Natural History. April, 1891.

Annual Report American Museum Natural History. New York, 1891.

John Hopkins University Circulars. July, 1891.

Proceedings of the Linnean Society of New South Wales. Vol. v., pt. 4. Sydney, 1890.

Leopoldina amtliches organ der Kaiserlichen Leopoldino-Carolinischen Deutschen Akademie der Naturforscher, edited by *Dr. C. H. Knoblaugh.* Halle, 1890.

Il Naturalista Siciliano. Palermo, 1891.

Bollettino Scientifico. Pavia, 1891.

Bollettino dei Musei di Zoologia ed Anatomia comparata della R. Universita di Torino. Vol. vi.

Fauna. Vol. i. Nos. 1—2. Société des Naturalistes Luxembourgeois.

Proceedings and Addresses at a Sanitary Convention, held by Charlevoix, Michigan. Lansing, 1891.

Missouri Agricultural College Experiment Station. Field Experiments with Corn.

Action of Dead Bacteria in the Living Body. By Mitchell Prudden, M. D., and Eugene Hodenpyl, M. D.

UNITED STATES DEPARTMENT OF AGRICULTURE.

What is Forestry? By B. E. Fernow.

Report of the Area of Corn, Potatoes, and Tobacco, and Condition of Growing Crops. July, 1891.

Horticultural and Kindred Subjects. By William Saunders.

HATCH EXPERIMENT STATION OF THE MASSACHUSETTS AGRICULTURAL COLLEGE.

Number 13, Directions for the use of Fungicides and Insecticides.

Number 14, Fertilizers for Corn.

